# The lived experiences of work and health of people living with deaf-blindness due to Usher syndrome type 2

**DOI:** 10.1080/17482631.2020.1846671

**Published:** 2020-12-07

**Authors:** Mattias Ehn, Moa Wahlqvist, Claes Möller, Agneta Anderzén-Carlsson

**Affiliations:** a Faculty of Medicine and Health, Örebro University, Örebro, Sweden; b The Swedish Institute for Disability Research, Örebro University, Örebro, Sweden; c Habilitation and Health, Region Stockholm, Stockholm, Sweden; d Audiological Research Centre, Faculty of Medicine and Health, Örebro University, Örebro, Sweden; e The Swedish National Resource Centre for Deafblindness, Lund, Sweden; f University Health Care Research Centre, Faculty of Medicine and Health, Örebro University, Örebro, Sweden

**Keywords:** Deafblindness, interpretative phenomenological analysis, Usher syndrome type 2, qualitative study, work

## Abstract

**Purpose**: This study aimed to explore lived experiences with working life from the perspective of people with deafblindness due to Usher syndrome type 2 (USH2).

**Background**: A limited number of studies have explored working life of people with Usher syndrome. One study of individuals with USH2 showed that work active reported significantly better psychological health compared to non-working individuals.

**Methods**: Seven participants aged 38–50 years with USH2 participated in interviews analysed by interpretative phenomenological analysis.

**Results**: The analyses yielded four themes showing that work is a *source of satisfaction* and a *commitment that needs to be balanced*. It is also associated with *facing limitations* and *feelings of uncertainty*.

**Conclusion**: Based on the psychology of work model we have demonstrated that work is associated with social connectedness, self-determination and a source of improved health outcomes. There are however also potential health hazards in people with USH2, indicating a need for balance between individual needs and resources, and an adapted environment, for maintaining or regaining health for actively working people with USH2.

## Introduction

This study explored lived experiences with working life from the perspectives of people with deafblindness due to Usher syndrome type 2.

The relation between work and health has been extensively studied in the general population, and Blustein (2008) concluded that work is one of the most important resources for meeting the fundamental psychological and social needs of individuals of working age. Work can fulfill the need for economic stability, status and power and mediates social connections with others through relationships and attachments (Blustein, 2008). Work also fulfils the need for self-determination, which represents what Deci and Ryan (2008) described as an important aspect of inner motivation that includes autonomy, competence and relatedness. While work can fulfill many fundamental needs, it is important to find a balance between work and leisure time, as health issues are more common among persons who report a poor work-life balance (Lunau et al., 2014). Job strain, tiring working conditions, and temporary job contracts are strongly associated with more reported health problems (Bambra et al., 2014). Other factors, such as moderate working duration and state welfare support, have been shown to be protective for maintaining work-life balance (Lunau et al., 2014). The greatest health risks are found outside the labour market, where a meta-analysis found that unemployment is a cause of poor health (Paul & Moser, 2009). Unemployment also increases the risk of morbidity and early mortality (Bambra & Eikemo, 2009; Dorling, 2009).

The labour market can be challenging for persons with disabilities, and reports by the Organization for Economic Co-operation and Development (OECD) show that persons with disabilities often rely on disability benefits (OECD, 2009a, 2009b). In Sweden, 12% of people of working age reported a disability, and among them, two-thirds reported a reduced work capacity (Statistics Sweden, 2019). Only 60% of people who reported a disability were employed compared to 80% in the general population, and while employment rates have increased during the last five years in the general Swedish population, they have remained steady among people with disabilities (Statistics Sweden, 2019). Individuals who are actively working are associated with a higher quality of life, a more positive attitude towards disability and higher self-efficacy compared with persons with disabilities who are unemployed or retired (Martins, 2015).

Although working life seems to be beneficial for people with disabilities, recent reports focusing on people with single sensory loss, such as hearing or visual impairment have also revealed challenges. A review of the literature on the working life of people with hearing impairment showed that high levels of stress and fatigue are common (Punch, 2016). Individuals with visual impairment reported difficulties in finding and maintaining employment (Benoit et al., 2013), and they experienced prejudice and encountered daily assumptions about their disabilities by colleagues and employers (French, 2017). In addition to the issues with attitudes, people with visual impairment experienced problems finding support and had difficulties travelling to work. Despite these barriers, one study showed that people with visual impairment remained resilient and were able to achieve their work-related goals of initial and sustained employment (French, 2017).

The combination of severe vision and hearing impairments constitutes a distinct disability that is defined as deafblindness (Nordic Centre for Welfare and Social Issues, 2019). The dual sensory loss affects face-to-face interactions and the ability to move around safely and restricts access to information, leading to limited activity and participation (Möller, 2008). Among the different syndromes that cause deafblindness, Usher syndrome (USH) is the most common. USH affects vision, hearing and, in some cases, vestibular function (Möller, 2003). USH is an autosomal recessive condition with an estimated prevalence of 6–10 per 100,000 individuals (Kimberling & Möller, 2013). USH is commonly divided into three subgroups, USH 1–3, based on clinical differences in the degree of hearing loss and problems with vision and balance (Millan et al., 2011). This study focused on people with Usher syndrome type 2 (USH2) who have congenital moderate-to-severe hearing loss and retinitis pigmentosa (RP), which is an eye disorder with progressive retinal degeneration. The progressive vision loss leads to severely impaired vision (Kimberling & Möller, 1995), where adults show low adaptability to light, night blindness, a restricted visual field and, eventually, impaired visual acuity (Hartong et al., 2006). USH2 is the most common form of USH in most countries (Leijendeckers et al., 2009; Sadeghi et al., 2006). In addition to the restrictions in hearing and vision, one study showed that people with USH2 report additional psychological and physical health problems (Wahlqvist et al., 2013). In other reports, high levels of stress were reported (Högner, 2015) in addition to a low quality of life, which was associated with a lack of social support (Dean et al., 2017).

The working life situations of people with USH, including those with USH2, reveal that stress is often related to the individual’s working life (Högner, 2015). A British interview study highlighted the limitations in employment options, a need for revising careers, and the fact that people with USH are often seen as not fit to work (Ellis & Hodges, 2013). Furthermore, their working life was described as tiring due to the constant effort to see and hear, and access to support was often described as unequally distributed. However, the study also showed the potential for these individuals to adapt to working life through methods such as part-time work or self-employment. Support from others and appropriate travel arrangements facilitated working life (Ellis & Hodges, 2013). In a previous study, we focused on life strategies for people with USH 2 and found that the participants valued opportunities to remain active and noted the important role that work and leisure activities played in their well-being (Ehn et al., 2019).

To the best of our knowledge, there is only one study that has focused on workinglife in relation to health measures for people with USH2. It found that people who were working reported significantly better psychological health compared to people who had a 100% disability pension (Ehn et al., 2016).

In summary, a limited number of studies have focused on the working life of people with a confirmed diagnosis of USH2. By exploring the lived experiences of people with USH2 with qualitative methods, such as IPA, we can provide a deeper understanding of their lived experiences with working life. This study could provide important insights that could serve as a basis for evidence-based guidelines for supportive and rehabilitative interventions.

### Purpose

This study aimed to explore lived experiences with working life from the perspective of people with deafblindness due to Usher syndrome type 2.

## Material and methods

### Design

This study utilized a qualitative explorative design (Patton, 2002) that focused on the lived experiences of the participants. The data were collected through semi structured interviews and analysed by interpretative phenomenological analysis (IPA) as previously described by Smith et al. (2009). IPA is an approach of qualitative inquiry, which aims at exploring how people make sense of their significant life experiences (Smith et al., 2009). Thus, it was regarded as an appropriate methodological approach relating to the aim of the present study. IPA is an approach that is:

- phenomenological (focusing on the experiences of a lived process filled with meanings which are unique for each individual, situated in the world),
- idiographic (concerned with an understanding of how particular phenomenon have been understood from the perspective of particular individuals, situated in a particular context) and
- interpretative (aims at facilitating and making sense of an appearance of a phenomenon).

IPA aims at conducting the examination of lived experiences in a way so that they are expressed in their own terms, rather than according to a pre-defined category system. According to Smith et al. (2009) this is what makes IPA phenomenological and related to the writings of traditional phenomenological philosophers.

### Participants and setting

Participants with USH2 were recruited in 2018 from a Swedish Usher research register that includes approximately 50% of the total USH2 population in Sweden. At the time of the study, 270 persons with USH2 across all ages were included in the register. The inclusion criteria were actively working people with a confirmed diagnosis of USH2 and being between 35–50 years of age. A purposeful sample of 14 participants was assembled that prioritized homogeneity, and all participants had a confirmed diagnosis of USH2. All the participants had moderate-to-severe hearing loss but were still able to use verbal communication in an interview setting. Only participants who were actively working 50% time or more for the last three years were included in the study. In Sweden, 40 hours per week is regarded as full-time work. The inclusion criteria were intentionally narrow due to the small sample size that is commonly used in an IPA (Smith et al., 2009).

An invitation letter was sent out by surface mail that was accessible with large print. The participants were informed about the aim of the study and the approximate duration of the interview and that they could choose a place and time that would be convenient. A written informed consent was returned along with information about the participants’ preferred telephone or email address for further communication.

Of the 14 persons who were invited, six did not respond to the invitation, and eight agreed to participate. However, one did not meet the criteria for work activity and was excluded. Seven individuals (4 women and 3 men) were included in the study. The age range of this group was 38–50 years, with a mean age of 42 years. The participants lived in different parts of Sweden and had engaged in work activity that ranged from 4 to 50 hours per week, with a mean of 28 hours a week. During one of the interviews, one participant disclosed a current work activity that did not meet the inclusion criteria (4 hours/week). However, this situation was only temporary, and the participant had recently had a higher level of work activity. Thus, the participant’s interview was included in the dataset. All participants had an upper secondary school education, and two have earned a college degree. All participants had white collar jobs, and administrative work was the most common position. All employments included digital, telephone or face-to-face interactions with colleagues or external contacts. All the participants had a specific workspace at an office, but some also had the opportunity to work from home. One of the participants was employed hourly, while the others were employed at permanent posts. None of the participants had a supported employment, but one had personal support that was subsidized by the government. Travel time to work ranged from 10 to 75 minutes, and most participants used public transportation. One walked, one used taxi services, and the others used a combination of cycling and public transportation.

All participants used hearing aids or had a cochlear implant, and five used a range of microphone systems or hearing loops in conference settings. Vision was improved for some individuals by tailored illumination that increased the contrast and accessibility of computers through special settings or through magnifying or specialized screen reader programs. Most participants also used spectacles or contact lenses on a daily base.

### Data collection

Prior to the interview, the participants were asked to answer a short structured and questionnaire that covered background information on education, type of employment, work activity, and years employed as well as information about the use of technical aids and the receipt of labour market support. The main study data were collected through individual, in-depth semi structured interviews. The individual interviews enabled the participants to share their lived experiences in their own terms. In accordance with the literature (Smith et al., 2009), the focus was on the participant’s involvement and understanding and his or her attempts to find a sense of meaning of work.

The interview schedule (Table I) served as guide to focus the interviews, in which the aim was to obtain information about the participant’s lived experiences of working life on both a descriptive and analytic level. Probes were posed for clarification and to encourage more analytical reasoning. The interviewer also had the option to leave the schedule when the interviewee presented other themes that were relevant to the purpose of the study, which was in accordance with previously described method (Smith et al., 2009).

**Table I.** Interview schedule

Question area	Main questions
Hearing and vision	Tell me about your hearing/vision impairment
	Please tell me how this affects your situation
	Do you have any other illness or impairment?
Employment/Work experience	Please, tell me about an ordinary day at work.
	What do you do at work?
	What is the best with your work?
	What can be difficult?
Health can affect work and work affect health	What are your experiences?
	How does your work affect your health?
	How does your health affect your work?
	What are the challenges?
	What are facilitating factors for you?
Follow up questions and probing questions	We have been talking about …
	What do you think about that?
	What does that mean to you?
	What emotions does this awake in you?
	What are the consequences for you?
	Could you please, reflect further on that …
	Could you, please elaborate on …
Final question	Is there anything that you think we should have been talking about that we have not yet talked about?

The interviews were conducted verbally in Swedish. Three interviews were conducted at the participant’s workplace, two were held at counselling and support sites for people with deafblindness, one was held in a home, and one was conducted at a research centre. The first author conducted all the interviews, which were audio recorded and ranged for 53–94 minutes with an average of 84 minutes. Some participants used a hearing loop together with their hearing aids to improve the sound quality during the interview. The settings were carefully chosen to reduce noise and dazzle and were configured to ensure proper illumination.

### Data analysis

The interviews were analysed by IPA (Smith et al., 2009). The recordings were transcribed verbatim and read through by all authors, and the audio recordings were repeatedly reviewed by the first author. Each interview was first analysed separately and divided into general and more specifically detailed parts where the authors separately made notes, exploratory comments, on any relevant parts of the transcript in order to explore how each participant understood, talked and reflected about their experience. In this step, the authors stayed close to the participant’s explicit meaning. This was followed by separate notations on a contextual level adding interpretation, focusing on the participant’s understanding, through involvement of the authors’ own pre-understanding. These comments were then discussed by the authors. In the third step, the authors left the transcript and focused the interrelationships, connections and patterns between all exploratory notes. This was when the authors understanding of the notations were used to identify emerging more abstract themes. The identified themes were first arranged in chronological order for each interview. In the following step, sections of each interview along with emerging themes were charted in a separate table for each interview. Clusters based on patterns with higher order “superordinate” themes were arranged, in accordance with the descriptions provided by Smith et al. (2009). Finally, the themes and superordinate themes derived from all individual analyses were compared and linguistically revised to make them more generic. All superordinate themes and themes that derived from the analysis were continuously evaluated in an iterative process focusing both the general and the particular.

The interpretative process of IPA was facilitated by the authors’ diverse range of professions and experiences, which created an active environment for reflection. The first author (ME) has 15 years of clinical experience with patients with USH. CM is a professor in audiology with 30 years of clinical experience and has extensive research experience with USH. AAC is an RN and an associate professor who specializes in qualitative methods and who has been active in research on deafblindness for several years. Finally, MW is a social worker and a Ph.D. who has researched USH and health and worked with people who have hearing loss, deafness and deafblindness.

### Ethical considerations

Prior to the interviews, all participants received written assurance that their participation was voluntary and that they had the right, without giving any reason and at any time, to suspend their participation. All participants signed an informed consent. Before starting the interviews, the purpose of the study and the voluntary nature of participation was repeated to the participant to avoid any misunderstanding of the written material due to vision impairment. The study was approved by the Ethics Committee of Uppsala Nr. 2012/515, 2012/515/2.

## Results

The interpretative phenomenological analysis of the seven individual interviews yielded four superordinate themes derived from 11 themes. For an overview, see Table II

**Table II.** Overview of the superordinate themes and themes

Superordinate themes	Themes
Feelings of satisfaction	Sense of belonging
	Feeling recognized
	Contentedness with one´s competence
A commitment that needs balancing	Taking responsibility for recovery
	Accepting the need for adaptations
	Prioritizing work tasks
	Making every effort
Facing limitations	Feeling exhausted
	Feelings of insufficiency
Feelings of uncertainty	Feeling insecurity on daily basis
	Perceptions of an unpredictable future

### Feelings of satisfaction

The sense of belonging in the workplace and the feeling of being appreciated and recognized as an important and competent member of the work force gave the participants a deep feeling of satisfaction. This could at times compensate for the difficulties associated with deafblindness, increase self-esteem and serve as an inner motivation to maintain the participants’ working life.

#### Sense of belonging

A sense of belonging to a group and being a part of a community was important for the health and wellbeing of the participants. It involved being identified by an employer and participating in a pleasant work group in a specific workplace or being a member of a specific profession. Work was regarded as an important arena for fulfiling the need for social interaction, and for some, this was the only place where they had close relationships with others outside their families. One participant revealed his experience with a previous work group:

We knew each other … since we have been working together a very long time. We could talk about everything … That was fun … they are nice … … a bunch of guys who have been there for a long time … … we were 15 persons who have been around a long time. We started to even go out and have a glass of wine or a beer or so … He added: … Well, I am that kind of person … my social life have been very much my work … not that much leisure time … (p4)

Belonging to a work group involved important aspects of mutual trust in which the participants felt comfortable and safe. Examples of the feeling of belonging were represented by just sitting down together and sharing a laugh or chat over a cup of coffee. For a few of the participants, the pleasant feeling of being part of a group helped them to overcome their inner conflict between not disclosing their deafblind-related problems and gaining the trust needed to share their hearing- and vision-related problems with their co-workers.

### Feeling recognized

The feeling of being recognized by colleagues and leaders as a competent member of the work force despite the difficulties caused by deafblindness provided a sense of satisfaction and self-confidence. This recognition could entail a co-worker asking for one’s opinion or advice or acknowledgement from an executive. It was also exemplified as being accounted for, e.g., when one’s colleagues would wait for the individual during business-related outings or when the work group counted on the success of the individual, which made the individual feel respected. Some participants highlighted that their colleagues or managers respectfully asked to schedule a meeting for a time that they knew could be inconvenient for the individual with deafblindness.

… If the meeting is in the afternoon, then she (the executive) always asks: NN, is it ok to have this monthly meeting in the afternoon? I can then check in my calendar, and then I say ‘yes, it is ok’. Then, I work at home the day after. (p1)

Managers or colleagues could also ask how to best adjust the workplace setting to facilitate the active participation of the person with deafblindness. In the home, the participants felt better recognized by their families when actively working. They emphasized that by being active workers, they were recognized as good role models who contributed to the family’s economic stability.

### Contentedness with one’s competence

A feeling of being content with their own competence was emphasized by all the participants. It was associated with improved self-confidence and a positive self-image, which could be threatened by deafblindness. Contentedness was also associated with the participant believing that he or she was the right person for the job, being creative and feeling capable of handling work assignments. These feelings extended from being able to use their skills, analytic abilities, and experiences in established, well-functioning routines. Sharing their own knowledge with colleagues to educate or support them was also highlighted by participants and was a source of satisfaction.

It is like this … … It is a comfort to experience that you are needed. That you have this knowledge … or, I really would like to continue my work. I want to help others and to be able to use the knowledge and experience I have. (p1)

### A commitment that needs balancing

Work had physical, cognitive and emotional costs affected by the deafblindness and could become a health hazard. By taking on responsibility for recovery, accepting the need for adaptations, prioritizing work tasks and adding a great effort, the participants strove to find a balance between work and their personal health.

### Taking responsibility for recovery

Recovery was crucial to finding enough energy to maintain a working life while balancing family and leisure time. The participants emphasized that work caused strain, stress, and tension and that it drained their energy levels. They took active steps to maintain a healthy work-life balance to ensure recovery. For some participants, a demanding work environment was balanced with exercise, social activities or spending time alone, which reflects how the definition of recovery was specific to the individual. A powernap could be revitalizing for some, while others remained physically active by taking daily walks or going to the gym to maintain their health and energy for work. *“Yes, I needed this. I am pretty sure that if I had not started physical exercises … then, then I would have … I would not have been seated here, that’s what I can say.”* (p7) Others emphasized that shifting their focus by spending time with friends, playing with their children or singing in a choir was a form of recovery for them.

### Accepting the need for adaptation

Working life presents individuals with deafblindness with many scenarios that can make work assignments difficult to perform. Being adaptable while managing the challenges of deafblindness was important for maintaining employment and for addressing obstacles that could otherwise lead to poor health outcomes or work performance. For some, actively identifying strategies to compensate for hearing or vision loss and accepting the need to take the initiative regarding these adaptations was associated with feelings of competence and relief.

At times, managers would force workplace adaptations that the individuals themselves did not identify or initiate. These actions were initially met with mixed feelings due to a sense of being controlled, but once positive experiences were associated with these changes, the negative feelings became feelings of thankfulness, relief and increased self-confidence. However, by accepting these adaptations, the individual was at risk of being seen as different by the other employees and of experiencing detachment from the work group. As a result, these adaptations were also associated with a sense of stigmatization. In some cases, the need for adaptations was facilitated through open and sincere communication with colleagues or managers. Furthermore, specialized support teams for people with deafblindness were valuable. It was also easier for individuals to accept these adaptations when they were regarded as prerequisites for continued employment.

A range of adaptations were highlighted among the participants. For example, reduced working hours, reduced workloads, alterations to work tasks and flexibility in how to perform assignments helped the participants to continue their work without unacceptable strain. The use of accessibility aids such as lighting adjustments, computer screen readers, or adapted office environments in addition to the adapted work tasks helped individuals remain active at work despite poor hearing and vision.

I require lots of light. That is, for example, why this room is extremely bright. But this is because I want it like this … really poor lighting, then it is extremely difficult to distinguish and to read etc … Then, I realized that I could have this room instead because it is much larger. Because I must sit facing the door as I am sitting now. (p2)

In some cases, economic subsidies were required to persuade employers to implement these adaptations at work before the participants could benefit from them. In some situations, such as staff meetings, communicative and sensory support were provided by an interpreter using tactile sign language.

### Prioritizing work tasks

Due to energy limitations and the difficulties associated with deafblindness, there was a need to prioritize certain work tasks and to balance work and leisure activities. Dedicating energy to one activity implied a need for compensation in another aspect of life by, for example, skipping a leisure activity to maintain health. Work was a high priority for many of the participants, which led to lower prioritization of leisure activities and family life in order to avoid risking one’s health. This was done out of a desire to do one’s duty or to maintain a satisfactory work performance, which helped the participants to feel content with their achievements. Some of the participants scheduled their work hours during times of the day when they felt alert and attentive and best able to use their limited vision and hearing. However, prioritizing work over family life resulted in feelings of guilt and shame for not being a good enough parent. In contrast, others described more positive feelings of self-confidence that were associated with contributing to the family’s economic stability.

I only work before noon because my eyes are most alert at that time. I have woken up after a good night’s sleep. So, if I have slept enough hours then I feel well the day after and am alert all morning, and then I feel that it is good that I am working, I … can be at my very best. (p1)

### Making every effort

By making every effort when performing work tasks, the participants strove to compensate for the limitations and challenges of deafblindness while attempting to achieve work results that were as good or better than those of their peers. This effort led to feelings of fulfilment and confidence but came at the cost of physical and psychological strain. This extra effort was seen as necessary due to the fear of insufficient work performance and was associated with the participant feeling that he or she was an unsatisfactory employee. The demand for extra effort was sometimes self-initiated but could also be attributed to external demands or comparing one’s own results to those of colleagues. It entailed spending extra time with visually demanding work tasks and, for some, included working extra hours outside the normal workday.

… I … when I can’t read fast anymore, then everything takes much longer. When I have to write, then it won’t turn out well, and I have to correct a lot. So, I believe that … if you compare to someone like, someone else who writes, then I probably need four times longer … everything takes more time (p7).

Despite headaches and constant fatigue, it was not obvious to the participants that they should alter or reduce their work activity. A sense of commitment, stubbornness and the denial of symptoms enabled the participants to continue working. Another aspect of making every effort was to actively take charge of team meetings to maintain control over communication, despite being restricted by poor hearing and vision. By taking charge, the participants felt that they were actively participating, but this was associated with loss of energy.

Although deafblindness often constrained the participants’ activities in their private and working lives, the opposite was also described. Work, at times, enabled them to extend their limits and take on challenges. These experiences often led to conflicting feelings of delight and fear. Taking on challenges was associated with positive feelings about being an individual who could still meet these demands, though these efforts often resulted in the individual feeling fatigued for one or more days after. By leaving one’s comfort zone, i.e., the comfortable office, and attending external meetings in unfamiliar environments, the participants reported increased self-esteem and increased confidence in their work role.

This results in an opportunity to meet these people face-to-face instead of just speaking to them by phone. And that is very useful, that is why I value my boss, that she can get me out of this zone of comfort. In the house where I work, I know every pillar, stair, door and everything. But (pause), I get to meet new interesting people. (p1)

### Facing limitations

The demands of the work environment included situations where the adjustments made for deafblindness were no longer adequate, and this needed to be acknowledged by the individual. Despite satisfying work experiences and strong commitments to employers, there were still participants who had to face their increasing limitations either suddenly or gradually. These instances resulted in feelings of insufficiency or exhaustion, and facing their limitations was associated with negative thoughts and emotions, decreased self-esteem and motivation to continue working. It was therefore common to try to neglect or avoid these thoughts for as long as possible. However, when the participants finally accepted their limitations and stopped fighting them, they often experienced feelings of relief.

### Feeling exhausted

Feelings of physical and mental exhaustion were related to an unsustainable working environment over time. Physical and mental symptoms such as headaches, body pain and constant fatigue were common. Moreover, the symptoms were persistent and affected the participants to such a degree that they, in their spare time, could not engage in activities that they used to enjoy. This could entail losing interest in their family life or their social and leisure activities. Such a situation could eventually lead to negative feelings and depression. The gradual development of fatigue was not easily identified at first, but in retrospect, after the participants regained their health, it was described as being trapped on *“a black tread mill.”* (p1) Another participant said,

You just can’t cope. You can’t engage in leisure activities or anything. It will just be working. And then you have no life besides work. And then … I became more depressed and since … since … life is not … life is more than work. (p3)

### Feelings of insufficiency

An inability to manage either personal or external work demands was associated with feelings of insufficiency. These feelings were closely related to a strong commitment or sense of duty to doing one’s best and were exacerbated when the participants’ performance was deemed insufficient compared to that of colleagues without visual or hearing problems or to the participants’ past achievements. An emerging feeling of not being able to fully meet organizational demands was also associated with feelings of insufficiency. During these times, the participants had thoughts of “I ought to … ”, and although they logically understood that these limitations were attributed to their restrictions in hearing and vision, there was a constant feeling of inadequacy:

But then it is really stupid to feel that you haven’t mastered this 100%. Because I have an enormous sense of duty and want to do right. So, this has been the heaviest, through the years, to have a feeling that I am not doing everything I would like to and need to do. (p5)

### Feelings of uncertainty

While working life was a source of satisfaction, restrictions in vision and hearing and constant visual deterioration led to daily feelings of uncertainty and a fear of an unknown and unpredictable future. The participants’ inability to anticipate what will happen or their feelings of insecurity during daily working life had negative impacts on their stability and security. Since working life was a platform for satisfaction, these feelings could negatively impact the participants’ commitments and self-image, and the associated uncertainty could lead to existential dilemmas.

### Feeling insecurity on a daily basis

Working with deafblindness leads to daily situations where restricted hearing and vision create moments of sensory or perceptual loss of control that are associated with feelings of insecurity. These moments often develop unexpectedly but can sometimes be anticipated based on previous experiences. Such anticipatory situations were associated with fear and feeling uneasy or anxious. Living with insecurity over time tends to increase arousal and can cause persistent tension. For example, in the office, someone might suddenly come up to the participant from behind while the participant is focusing on the computer, or a door might unexpectedly open while the participant is walking down a hallway, and he or she might not react in time: “*Before, I was sitting (if I was sitting) on one side in the room. Then I couldn’t see that you were entering, so I often got very surprised and scared when someone entered the room … ”* (p2).

Different strategies were used to reduce insecurity. Utilizing the sense of smell was one way of identifying environmental risks and potentially detecting someone approaching. Daily insecurity could also be partially mitigated by staying in one’s comfort zone. However, we previously highlighted the benefits of leaving one’s comfort zone for some individuals since the satisfaction derived from new experiences outweighed the costs of increased insecurity.

### Perceptions of an unpredictable future

A diagnosis of deafblindness and its anticipated progression could lead to an unpredictable future in the workforce and, by extension, in many other aspects of life for affected individuals. Our participants feared what would happen to their working life in the near or distant future. This led to the anticipation of being excluded from their work group and becoming detached from colleagues and friends. Furthermore, an inability to work threatened their identity and raised questions about their economic security, which caused distress, negativity, rumination, worrying and anxiety. The participants tried to regain control of negative thoughts or worries by focusing on the present rather than the unpredictable future:

It doesn’t feel like something positive. No. It feels like … somewhere I don’t want to be. But I want to be here now. I am very much like this: ‘No, what´s in the future, we have to deal with later.’ Then sometimes you can get very dark moments or what it is named. I think, what is going to happen? Will I manage by myself? Will I be able to do everything I can do today? But I try to ignore these thoughts; I am thinking, I am here and now. I have to focus on that. It is like … It took time for me to reach this far, so you can do that. In the beginning when the diagnosis was made, then of course, I was terrified and thought that I would be blind the next day. But this is not really the case. (p2).

## Discussion

This study aimed to explore lived experiences with working life from the perspective of people with deafblindness due to Usher syndrome type 2. In the following sections, we summarize the results of this study and discuss how they relate to other studies of people with USH or deafblindness and the relationship to a psychology of work model that emphasizes the role of work as a fundamental human need (Blustein, 2008). Furthermore, we briefly discuss the results in relation to the Meikirsh holistic health model (Bircher & Kuruvilla, 2014), which defines health as:

… a state of wellbeing emergent from conducive interactions between individuals’ potentials, life’s demands, and social and environmental determinants. Health results throughout the life course when individuals’ potentials – and social and environmental determinants – suffice to respond satisfactorily to the demands of life. (Bircher & Kuruvilla, 2014, p. 4)

In reviewing the results of this study, we show a pattern that demonstrates the importance of feeling satisfied at work, which includes a sense of belonging in the workplace and being appreciated and recognized as an important and competent member of the workforce. In turn, this motivated the participants to maintain their role in working life. However, work was also a commitment that was associated with health hazards, which needed to be balanced. By taking on personal responsibility for their own recovery, accepting that adaptations were necessary, prioritizing work tasks and making extra efforts, the participants strove to achieve balance. The demands of working life were also associated with the participants facing their own limitations. Working life elicited feelings of inadequacy and led to exhaustion that was associated with decreased self-esteem and motivation to continue to work. Feelings of work-related uncertainty were also associated with the progression of deafblindness. These uncertain feelings led to feelings of insecurity on a daily basis and a sense of the unpredictability of working life in the future, which threatened the participants’ sense of stability and identity.

A previous study from our group on the psychological and physical health of people with USH2 showed that there were significantly fewer health problems among actively working individuals than among non-working people (Ehn et al., 2016). This study was based on a public health questionnaire of reported health issues and demonstrated that people with USH2 who were actively working reported less severe effects regarding losing faith in themselves, viewing themselves as worthless and reporting difficulties in handling problems when compared with nonworking participants (Ehn et al., 2016). This is similar to our present findings, which reveal that actively working people with USH2 exhibit improved confidence and feel valued and feel competent in handling work-related problems. The connection between work and feelings of competence, belonging and recognition was previously described in a British USH-population by Ellis and Hodges (2013), where experiences, including working life, were studied. However, in the Ellis and Hodges (2013) study, the participants had different clinical types of USH, and most participants had a supported form of employment within different disability organizations.

The satisfaction and sense of belonging related to work that we show in our study can be interpreted through the psychology of work model (Blustein, 2008), in which people with USH2 fulfill fundamental needs for social connectedness and self-determination through work. Work also forced the participants to face their own limitations, which was also previously shown by Ellis and Hodges (2013). In addition, Högner (2015) found that high levels of stress were related to work, indirectly indicating a strenuous work environment for people with USH.

Fatigue and stress were previously reported as the most prominent health problems among people with USH2 (Wahlqvist et al., 2013), and stress and fatigue were found to be common in both actively working and nonworking people with USH2 (Ehn et al., 2016). This indicates that stress or fatigue may be related to other factors outside of working life. However, the impact of work as an important source of fatigue should not be underestimated. Several studies in people with hearing loss have shown that fatigue is common in working life (Hua et al., 2015; Punch, 2016; Svinndal et al., 2018). The study by Hua et al. (2015) showed, in agreement with our study, that exhaustion and fatigue reduced participants’ ability to take part in leisure activities and family life. Moreover, as in the Svinndal (2018) study, our findings show that exhaustion was sometimes associated with long-term sick leave.

The results indicate that in addition to the positive experiences of having a job, there is a risk of developing a poor work-life balance due to job-strains that can lead to fatigue. Lunau et al. (2014) stresses the importance of balancing work and leisure time and that people who perceive a poor work-life balance generally report increased health problems. On the other hand, moderate working hours and state welfare support have been shown to be beneficial for work-life balance (Lunau et al., 2014). This also appears to be true for the participants in the present study. By reducing working hours and accepting adaptations at work or prioritizing work tasks, the participants made a commitment to achieving a healthy work-life balance. They were well aware of the health hazards associated with deafblindness. In the context of Sweden, income subsidies, social insurance and support for adaptations in the workplace are all examples of societal support, and these were specifically mentioned in the interviews. Moreover, the commitment to balancing health risks found in this study was paired with the experience of feeling recognized and to feeling like one belonged in the workplace. Blustein (2008) showed that social connectedness is helpful when handling workplace challenges and for mitigating work-related stress.

Although the participants worried about their future place in the workforce, which they found unpredictable, our results also show that the positive aspects of work helped some of the participants to stay focused on the present instead of ruminating or worrying about an unknown future. Work as a source of focusing on the present was also found in a report of life strategies (Ehn et al., 2019), where work along with leisure activities were enjoyed and used as a way to appreciate the present. This was also shown in the Ellis and Hodges (2013) study, where the participants continued to strive towards their own career goals regardless of what the future might bring.

The impact of uncertainty regarding how long the participants will be able to continue to work can be understood in the context of work being a fundamental human need, as stated by Blustein (2008). He stressed that work is related to survival and self-determination. In the present study, the fear of not being able to work was associated with a fear of poorer economic stability, reduced status and loss of power. Moreover, risking one’s future autonomy, competence and relatedness due to the need to stop working was related to the fundamental need for self-determination (c. f. Blustein, 2008).

The findings of the study indicate that work both has positive and negative health outcomes, which can be interpreted in a broader biopsychosocial health perspective. The experiences from working life mirrors that a balanced working life for people with USH2 not only fulfill fundamental psychological needs (Blustein, 2008), but seems to play an important role for their overall health and well-being. In the Meikirch model of health (Bircher & Kuruvilla, 2014), health occurs when an individual’s biologically given and acquired potentials, along with social determinants suffice to respond satisfactorily to demands of life (Bircher & Kuruvilla, 2014). This is an ongoing process that continues through life, since determinants as well as the demands of life are not static. The results in the present study indicates that when the demands of working life are met by the person with USH2, a situation of balance is achieved which affect health in a positive way. The participants’ use of their own resources (i.e., acquired potentials), such as using skills and social connectedness, are vital and might even buffer for restrictions in hearing and vision abilities (i.e., biologically given potentials). A prerequisite is however, the presence of an adapted environment, inclusive norms, values and support from the work place as well as the society. The use of a health model such as the Meikirch model provides new insights on the importance of work for maintaining health for persons with USH2. It offers an understanding of the importance of a lifelong ongoing process to find a balance between individual needs and resources, and an adapted environment, for maintaining or regaining health for actively working people with USH2.

### Strengths and limitations

Due to the limited research on people with USH2, a qualitative explorative design was employed, and a phenomenological approach seemed appropriate for exploring lived experiences with work and health in people with USH2. An interpretative phenomenological analysis matched the rather small number of participants and enabled the accumulated knowledge of the authors to be used as a natural part of the interpretation process. Although focusing on the particular covering the experiences of seven individuals, each case cannot be described in such detail as would have been possible with fewer participants. In such cases, what still makes it an IPA inquiry is that the results are illustrated with particular examples from the individuals’ narratives and that themes reflect recurrent pattern of experiences identified in the data (Smith et al., 2009). The use of a semi structured interview guide with the first author conducting all interviews enhanced the dependability and credibility of these data. However, we are cognizant that the dependability of the data might have been affected in the later interviews by the knowledge obtained during the earlier interviews. However, confirmability and credibility were enhanced through audio recordings and professional transcriptions as well as the participation of all authors in the analysis (c.f Smith et al., 2009). The transferability of the qualitative results is best evaluated by the reader in light of the background data.

## Conclusions

A limited number of studies have explored working life of people with Usher syndrome. The findings of this study provide new insights about the lived experiences of people with USH as it highlights the satisfaction and sense of commitment that extend from fulfiling the desire to find a healthy balance between work and other aspects of life. However, these experiences demonstrated particular challenges related to working and living with deafblindness involving a progressive visual impairment. Most of the experiences identified in this study are in line with those of the general population, where work has the potential to fulfill the fundamental human needs for survival, social connectedness and self-determination as described in the psychology of work model. The study stresses the significance of work for health and well-being in people with deafblindness due to USH 2, but also the potential health hazards of a group of people with USH2 that do not have a balanced working life. The particular challenges and health hazards found in the USH2 population highlight the importance of taking time for recovery, addressing adaptations in the work environment and receiving recurrent state services.

## Implications for practitioners and policy makers

The results of this study provide new insights that could be used by professionals to initiate a dialogue with people with USH to maintain or regain a balance in working life. The present study shows that people with USH2 want to use their resources in working life, despite hearing and vision problems, and that work is an important area to address in rehabilitation and counselling. The experiences of people with USH2 further stresses the importance for policy makers to continue make every effort to include people with deafblindness in working life to promote health and well-being, and to facilitate for individual adaptations. Studies are needed to address the situation of, and develop support tailored for persons with USH2 who have left or never have had access to the labour market.
